# Predicting measles elimination through surveillance-response and health system determinants: a village-level structural equation modeling study in Cirebon Regency, Indonesia

**DOI:** 10.1016/j.ijregi.2026.100986

**Published:** 2026-08-28

**Authors:** Agus Salim, Hari Basuki Notobroto, Fariani Syahrul, Ade Nurlina

**Affiliations:** 1 Doctoral Program of Public Health, Faculty of Public Health, Universitas Airlangga, Surabaya, Indonesia; 2 West Java Provincial Health Office, Bandung, Indonesia; 3 Department of Epidemiology, Biostatistics, Population Studies, and Health Promotion, Faculty of Public Health, Universitas Airlangga, Surabaya, Indonesia; 4 Cirebon Regency Health Office, Cirebon, Indonesia

**Keywords:** Measles elimination, Surveillance-response, Structural equation modeling, Systems epidemiology, Village-level surveillance

## Abstract

**Objectives:**

Measles elimination remains a major public health challenge despite the widespread availability of effective vaccines. Rising outbreaks in some countries suggest that high immunization coverage alone may not be enough to stop transmission. However, evidence remains limited regarding how surveillance-response, health system capacity, nutritional status, and community vulnerability interact to influence measles elimination within a single epidemiological framework. This study aimed to develop and validate an integrated epidemiological prediction model for measles elimination using structural equation modeling–partial least squares (SEM-PLS) at the village level in Cirebon Regency, Indonesia.

**Methods:**

A cross-sectional study was conducted using secondary data from 424 village administrative units in Cirebon Regency during 2022-2024. Variables included measles immunization coverage, nutritional status, village vulnerability, health resources, surveillance-response performance, suspected measles cases, confirmed measles cases, and transmission incidence. Data were analyzed using SEM-PLS to estimate the direct and indirect pathways linking surveillance-response, immunization, nutritional status, health system capacity, community vulnerability, and transmission to measles elimination.

**Results:**

The SEM-PLS model explained 56.9% of the variance in measles elimination. Surveillance-response performance emerged as the principal mediating mechanism linking health system capacity with reductions in suspected cases, confirmed cases, and transmission. Measles elimination was significantly associated with confirmed cases (*P* < 0.001), suspected cases (*P* < 0.001), immunization coverage (*P* = 0.002), nutritional status (*P* = 0.003), village vulnerability (*P* = 0.001), health resources (*P* = 0.001), and surveillance-response performance (*P* < 0.001). These findings indicate that surveillance-response contributes substantially to elimination through early case detection and interruption of transmission pathways.

**Conclusion:**

Measles elimination should be viewed not solely as a vaccination outcome but as the product of an integrated epidemiological system in which surveillance-response, health system capacity, nutritional status, and community vulnerability interact to interrupt transmission. The proposed predictive model provides a practical framework for evidence-based elimination planning and more adaptive, evidence-informed measles elimination policies in endemic settings.

## Highlights

• A systems epidemiology model for measles elimination was developed and validated.
• Surveillance-response outperformed immunization as the central mediating pathway.
• Health resources, nutrition, and community vulnerability jointly influenced elimination.
• The structural equation modeling–partial least squares model explained 56.9% of the variance in measles elimination.
• Findings support integrated, village-level strategies for measles elimination in endemic settings.

## Introduction

Measles remains a major vaccine-preventable disease, with outbreaks driven by immunization disruptions, population mobility, and immunity gaps, thereby hindering elimination efforts. Measles and rubella elimination is defined as the absence of endemic virus transmission in a defined geographical area with a well-performing surveillance system. Formal verification requires at least 36 months without endemic transmission, supported by high-quality surveillance and genotyping evidence [1]. Even though vaccines have greatly helped to reduce disease-related mortality, some countries are seeing a resurgence in cases, which underscores the importance of improving community protection and disease surveillance [[2], [3], [4]]. In this context, measles elimination should be understood as a multilevel process in which achievements in immunization, surveillance, and outbreak response at the village level provide a foundation for progress at the regency/city and provincial levels, ultimately supporting national elimination goals.

Although measles elimination is assessed at the national level, its verification relies on evidence generated through surveillance and program implementation across subnational levels. The World Health Organization (WHO) South-East Asia strategy emphasizes achieving and maintaining high population immunity and sensitive case-based surveillance across all areas and districts within each country [5]. In Indonesia, surveillance performance indicators are also specified at the regency/city level, including a discard rate of ≥2 per 100,000 population, 100% laboratory confirmation of suspected measles cases, and timely epidemiological investigation. National evidence for elimination is subsequently reviewed through the regional verification process [6]. Therefore, achieving and maintaining elimination standards at the village, regency/city, and provincial levels is important for demonstrating and sustaining national measles elimination.

Indonesia still has a substantial measles burden, especially in West Java Province, where Cirebon Regency has experienced numerous outbreaks and a high number of suspected cases. The regency includes urban, semi-urban, and rural areas, each with different population characteristics, access to healthcare, effectiveness of immunization programs, and health surveillance capacity. This diversity leads to significant differences in community vulnerability and disease transmission, which underscores the important of using detailed models at the regency level to identify areas at higher risk, areas that might be overlooked when using aggregate statistics [7].

Measles elimination relies on many factors working together, not just on vaccination coverage. Even with high vaccination rates, factors such as weak immunity, delayed vaccination, and imported cases could allow the disease to continue spreading [8]. Good surveillance and response systems are important for detecting cases early and controlling outbreaks [2,9]. In addition, high population density, poverty, population mobility, malnutrition, and inadequate health resources make a community more likely to be affected by measles outbreaks [3,10].

Recent evidence shows that measles elimination should be seen as a complex issue involving multiple components of the health system, rather than simply as a matter of vaccine administration [3]. Most earlier research has focused on vaccination rates, outbreak descriptions, or temporal patterns, but has not integrated surveillance performance, nutritional status of people, available health resources, community vulnerability, and indicators of disease transmission into a single system [11]. Because there is limited evidence regarding the effectiveness of integrated approaches to measles elimination, especially in poorer countries, the evidence base remains insufficient [8,12].

Structural equation modeling (SEM) enables the simultaneous evaluation of direct and indirect relationships among multiple determinants [12]. This approach is well suited to analyzing complex interactions between surveillance-response performance, immunization, nutritional status, health resources, community vulnerability, and disease transmission, thereby providing a stronger evidence for public health decision-making than analyses focusing on isolated factors [3,11].

Therefore, this study developed a village-level epidemiological prediction model for measles elimination in Cirebon Regency using structural equation modeling–partial least squares (SEM-PLS). The model integrates surveillance-response performance, immunization coverage, nutritional status, health resources, community vulnerability, suspected and confirmed measles cases, and transmission incidence within a single analytical framework. By applying a systems epidemiology perspective, this study provides evidence to support surveillance strengthening, resource prioritization, and targeted measles elimination strategies at the subnational level, particularly in high-burden settings in developing countries [4,13,14].

## Methods

This study employed a census-based ecological analytical design using routinely collected surveillance and public health data to develop a predictive model of measles elimination in Cirebon Regency, West Java Province, Indonesia. The unit of analysis was the administrative village, with all variables representing aggregated village-level indicators. Cirebon Regency was purposively selected because it consistently reported one of the highest numbers of suspected measles cases in West Java during 2022-2024. The theoretical population comprised administrative villages that implemented the Indonesian national measles surveillance and immunization programme, while the target and study populations included all 424 villages in Cirebon Regency. The sampling frame was the official village registry linked to the District Health Office surveillance database. Because all eligible villages were included, a complete census was conducted; therefore, no sampling procedure or sample size calculation was required.

Secondary data collected between January 2022 and December 2024 were obtained from surveillance, immunization, nutrition, demographic, and health resource information systems. Variables included immunization coverage, nutritional status, village vulnerability, health resources, surveillance-response performance, suspected and confirmed measles cases, and transmission incidence. The primary outcome, measles elimination status, was modeled as a latent construct based on indicators of sustained transmission interruption and surveillance performance, consistent with the WHO measles elimination framework (World Health Organization, 2013). A systems epidemiology framework was applied using SEM-PLS to simultaneously evaluate the direct and indirect relationships among latent constructs and identify key determinants of measles elimination [[15], [16], [17], [18]].

The study analyzed aggregated surveillance data without individual identifiers. Therefore, informed consent was waived. The study adhered to international ethical standards for epidemiological research involving secondary surveillance databases. Permission to access surveillance data and health system data was obtained from the relevant district health authorities prior to the start of the study [19].

### Variables and measurements

The study examined village-level determinants of measles elimination, including village vulnerability, health resources, nutritional status, measles immunization, surveillance-response, suspected measles cases, confirmed measles cases, and measles transmission. Village vulnerability was represented by population density and household poverty. Population density was calculated as the number of residents per unit area, while household poverty was represented by the proportion of households receiving direct cash assistance (*BLT*). Health infrastructure was defined as the number of hospitals, health centers, auxiliary health centers, private clinics, and Posyandu in each village. Health resources represented the number of doctors, midwives, nurses, and health cadres available in each village. Nutritional status was measured by the proportion of children with severe or moderate malnutrition based on weight-for-height. Measles immunization was measured as the coverage of children receiving two doses of measles-containing vaccine. Surveillance-response represented the performance of epidemiological investigation and specimen collection among reported suspected measles cases. Suspected measles cases were defined as the number of reported suspected cases, whereas confirmed measles cases referred to laboratory-confirmed cases reported during 2022-2024. Measles transmission was defined as the occurrence of two or more cases within a 7-day period in the same village.

## Results

### Characteristics of villages

Among the 424 villages, 176 (41.5%) failed to achieve measles elimination during 2022-2024. These villages generally exhibited higher numbers of suspected and confirmed measles cases, lower immunization coverage, and weaker surveillance-response performance, indicating heterogeneity in epidemiological vulnerability and health system capacity. Subsequent analyses identified the determinants of persistent measles transmission.

Table 1 presents the characteristics of the 176 villages that did not achieve measles elimination and were included in the analysis. During the 2022-2024 research period, these villages continued to experience measles transmission, with an average of 3.15 ± 2.27 confirmed measles cases, 7.55 ± 5.51 suspected measles cases, and 2.22 ± 1.62 transmission events. The average two-dose measles immunization coverage was 80.49%, while substantial variation was observed in nutritional status, population characteristics, health resources, and surveillance-response performance. These findings indicate substantial heterogeneity in epidemiological and health system conditions among villages that have not yet achieved measles elimination.

**Table 1:** Characteristics of villages that did not achieve measles elimination (n = 176).

Variable	Mean		SD	Min	Max
Measles cases	3.15	±	2.27	1	14
Suspected measles cases	7.55	±	5.51	1	31
Transmission events	2.22	±	1.62	1	9
Two-dose measles immunization coverage (%)	80.49	±	19.84	18.37	100
Prevalence of malnutrition (%)	11.55	±	5.54	0	28.9
Population with primary education (%)	46.99	±	8.62	15.51	64.71
Village area (km²)	2.62	±	1.95	0.18	12.78
Population size	6691.89	±	2438.00	2666	16,622
Population density (persons/km²)	3818.26	±	4383.49	436.88	54,505.6
Number of households	2155.86	±	859.11	900	5528
Poor households	62.36	±	65.52	0	557
Health budget allocation (%)	6.59	±	3.47	0	17.57
Health infrastructure	33.49	±	17.64	3	88
Health workers	32.88	±	18.10	2	100
Surveillance-response score	6.00	±	4.26	1	22

Abbreviation: SD, standard deviation.

### Evaluation of measurement models

Reliability and validity analyses confirmed that all constructs met the recommended SEM-PLS criteria. Composite reliability and Cronbach's alpha exceeded 0.70, average variance extracted values were above 0.50, and the Fornell–Larcker criterion confirmed adequate discriminant validity, supporting the suitability of the measurement model for structural analysis.

Table 2 shows that all latent constructs met the recommended reliability and validity criteria. Composite reliability indicated strong internal consistency, average variance extracted values exceeded 0.50, and all constructs were retained for subsequent structural model analysis. The structural model explained 56.9% of the variance in measles elimination (R² = 0.569), indicating moderate predictive performance. Confirmed measles cases and transmission incidence showed the strongest direct effects, whereas surveillance-response performance exerted substantial indirect effects by reducing suspected measles cases and transmission, highlighting its central role in linking health system characteristics with measles elimination.

**Table 2:** Construct reliability and validity assessment.

Construct	Cronbach’s alpha	Composite reliability	AVE
Village vulnerability	0.842	0.887	0.613
Health resources	0.861	0.901	0.646
Nutritional status	0.815	0.873	0.578
Immunization coverage	0.884	0.918	0.702
Surveillance-response	0.896	0.924	0.708
Suspected measles cases	0.803	0.864	0.561
Confirmed measles cases	0.821	0.879	0.595
Transmission incidence	0.839	0.891	0.621
Measles elimination	0.872	0.913	0.677

Abbreviation: AVE, average variance extracted.

### Direct effects on measles elimination

Table 3 shows that only confirmed measles cases (β = 0.206, *P* < 0.001) and transmission incidence (β = 0.197, *P* < 0.001) had significant direct effects on measles elimination. These findings identify sustained laboratory-confirmed measles transmission as the primary epidemiological barrier to achieving elimination.

**Table 3:** Direct effects on measles elimination.

Pathway	β	*P*-value
Confirmed measles cases → measles elimination	0.206	< 0.001
Transmission incidence → measles elimination	0.197	< 0.001

### Indirect effects on measles elimination

Table 4 summarizes the indirect effects of the study variables on measles elimination through intermediary pathways. The SEM-PLS analysis identified several significant indirect pathways to measles elimination, suggesting that elimination is shaped by the interaction of surveillance performance, health system capacity, immunization, nutritional status, and disease transmission dynamics. These findings support the conceptual framework that measles elimination is a systems-based public health outcome involving multiple interconnected epidemiological processes.

**Table 4:** Indirect effects on measles elimination.

Pathway	β	*P*-value
Suspected measles cases → measles elimination	0.288	< 0.001
Surveillance-response performance → measles elimination	0.219	< 0.001
Health resources → measles elimination	0.075	< 0.001
Village vulnerability → measles elimination	0.039	0.001
Measles immunization coverage → measles elimination	0.016	0.002
Nutritional status → measles elimination	−0.029	0.003

Among all indirect pathways, suspected measles cases demonstrated the strongest indirect association with measles elimination (β = 0.288, *P* < 0.001). This finding suggests that early identification of suspected cases may enable timely surveillance and response activities that interrupt subsequent transmission and improve the likelihood of achieving elimination. Villages that are capable of detecting suspected measles cases promptly are therefore more likely to implement timely investigation, laboratory confirmation, and response measures before sustained community transmission occurs.

Surveillance-response performance also exhibited a substantial indirect effect on measles elimination (β = 0.219, *P* < 0.001). Rather than influencing elimination directly, surveillance-response appeared to influence elimination indirectly by reducing suspected cases, limiting transmission, and ultimately decreasing laboratory-confirmed measles cases. This finding highlights the central mediating role of surveillance-response in linking health system performance to measles elimination.

Health resources (β = 0.075, *P* < 0.001), village vulnerability (β = 0.039, *P* = 0.001), and two-dose measles immunization coverage (β = 0.016, *P* = 0.002) also showed significant indirect contributions to measles elimination. Although these effects were smaller than those observed for surveillance-response, they indicate that an adequate health workforce, sufficient infrastructure, lower community vulnerability, and sustained immunization programs collectively strengthen the epidemiological conditions required to interrupt measles transmission.

Conversely, poorer nutritional status was associated with a significant negative indirect effect on measles elimination (β = −0.029, *P* = 0.003), suggesting that poorer nutritional conditions may reduce community resilience against measles transmission and indirectly hinder elimination efforts. Collectively, these findings indicate that successful measles elimination depends on coordinated improvements across surveillance, immunization, nutrition, and health system capacity rather than on a single intervention.

### SEM drawing

**Figure 1:**
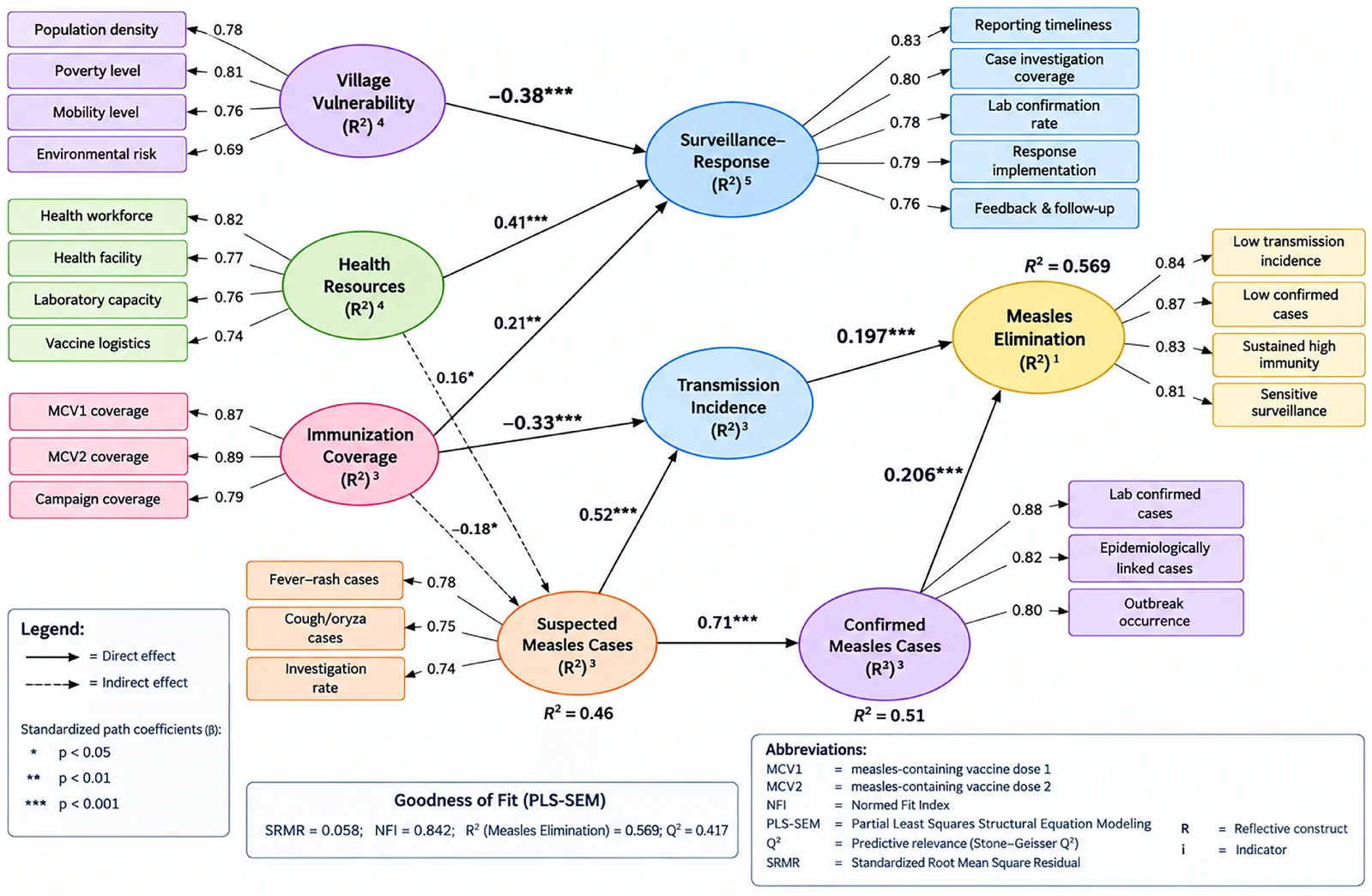
Structural equation model of measles elimination predictors.

Abbreviations: MCV1 = first dose of measles-containing vaccine; MCV2 = second dose of measles-containing vaccine; NFI = normed fit index; PLS-SEM = partial least squares structural equation modeling; Q² = predictive relevance; SRMR = standardized root mean square residual; β = standardized path coefficient.

Figure 1 presents the final SEM-PLS model describing the epidemiological pathways to measles elimination. Surveillance-response performance occupied a central mediating role, linking health resources, immunization coverage, nutritional status, and village vulnerability to disease transmission. Confirmed measles cases and transmission incidence were the principal proximal determinants directly influencing measles elimination, highlighting that elimination depends on coordinated interactions among surveillance quality, health system capacity, community vulnerability, and disease occurrence rather than vaccination coverage alone. The structural model demonstrated moderate predictive performance, explaining 56.9% of the variance in measles elimination (R² = 0.569). Overall, the findings support the proposed systems epidemiology framework, indicating that measles elimination is driven by interconnected epidemiological processes rather than isolated interventions.

## Discussion

The present study developed an integrated epidemiological model showing that measles elimination is shaped by interactions among surveillance-response performance, health system capacity, immunization coverage, nutritional status, village vulnerability, and disease transmission dynamics rather than by vaccination coverage alone. The SEM-PLS model explained 56.9% of the variance in measles elimination, highlighting the importance of considering elimination as a multidimensional systems process rather than the consequence of isolated interventions.

Among all determinants, surveillance-response performance emerged as the central mediator linking health system capacity with reductions in suspected cases, confirmed cases, and transmission incidence. This finding suggests that surveillance functions not only as a monitoring system but also as an active component of the public health response that can facilitate the interruption of transmission before outbreaks become established, consistent with evidence from countries that have sustained measles elimination through sensitive surveillance systems [20]. The indirect effect of health resources further suggests that adequate workforce capacity, laboratory support, and service infrastructure strengthen surveillance-response performance and, consequently, improve elimination outcomes. This finding supports previous evidence that resilient local health systems are essential for timely outbreak detection and sustained measles elimination in endemic settings [21].

Beyond health system capacity, population immunity remained an essential component of the elimination pathway. Although routine measles immunization was an important determinant, its effect was primarily indirect, indicating that high vaccination coverage alone is insufficient to achieve elimination without sensitive surveillance and timely outbreak response. These findings reinforce evidence that homogeneous population immunity, rather than aggregate vaccination coverage alone, is critical for sustaining measles elimination [22]. Village vulnerability was negatively associated with measles elimination because densely populated and socioeconomically disadvantaged communities, particularly those with high population mobility, may face greater risks of persistent transmission, limited health care access, delayed case detection, and lower immunization uptake. This finding underscores the importance of addressing structural and environmental determinants alongside immunization and surveillance in elimination strategies [23].

Nutritional vulnerability may indirectly affect measles elimination by increasing susceptibility to measles and, consequently, the occurrence of suspected and confirmed cases. Poor nutritional conditions can harm immune function and reduce resistance to infectious diseases, especially in children. Malnutrition has long been associated with greater severity of measles infection and an increased risk of transmission in communities experiencing socioeconomic disadvantage. The present findings suggest that interventions aimed at improving nutritional status may indirectly support measles elimination. This perspective broadens the conventional understanding of measles elimination beyond an immunization-centered approach [24].

An important finding of this study is the complementary role of suspected measles cases, laboratory-confirmed cases, and transmission incidence within the elimination pathway. Suspected case notifications function as an early warning indicator that enables timely epidemiological investigation, laboratory confirmation, and outbreak response, highlighting the importance of sensitive case-based surveillance for preventing the re-establishment of endemic transmission [25]. In contrast, laboratory-confirmed measles cases emerged as the strongest direct determinant of measles elimination, reflecting persistent virus circulation as the primary barrier to elimination and reinforcing WHO recommendations on the importance of laboratory-supported surveillance [26]. Together with transmission incidence, these indicators represent the cumulative effects of surveillance-response performance, population immunity, nutritional vulnerability, health system capacity, and community susceptibility, indicating that sustainable measles elimination requires coordinated improvements across surveillance, immunization, nutrition, and health system preparedness rather than reliance on a single intervention [27].

Beyond identifying individual determinants, this study demonstrates that measles elimination is a systems outcome driven by interactions among surveillance-response performance, health system capacity, immunization coverage, nutritional status, village vulnerability, and disease transmission. Unlike previous studies that examined these factors separately, the integrated SEM-PLS framework identified surveillance-response as the central mediator linking health system characteristics with reductions in suspected cases, confirmed cases, and transmission incidence. These findings advance the application of systems epidemiology to measles elimination and provide a practical framework for prioritizing integrated interventions and strengthening subnational elimination strategies [28].

The community-level heterogeneity represents another important finding that emerged from this study. Significant variation between villages suggests that district-level averages can obscure pockets of local vulnerability that may sustain transmission. Communities with lower immunization coverage and weaker surveillance systems may continue to hinder progress toward broader elimination despite improvements at the regional level. These findings support recent recommendations advocating for monitoring subnational elimination and strengthening village-based surveillance. Therefore, targeted interventions may be more effective than a uniform approach across villages or districts [29].

Predictive modeling using SEM-PLS provides an important methodological advantage for understanding the complexity of measles elimination pathways. Unlike conventional regression approaches, SEM allows for the simultaneous evaluation of the direct and indirect relationships among epidemiological, surveillance, and health system variables. The ability to assess interconnected pathways improves understanding of how vulnerability, healthcare capacity, and surveillance systems together affect elimination outcomes. This analytical approach represents an important methodological contribution to measles elimination research, particularly in low- and middle-income countries, where integrated systems-based epidemiological modeling remains limited. These findings highlight the potential value of SEM-PLS for supporting evidence-based measles elimination planning [30].

The integrated model developed in this study further reflects the complex systems nature of measles elimination. Measles elimination appears to result from a dynamic interaction between surveillance quality, immunity levels, healthcare capacity, and community vulnerability rather than from isolated determinants alone. These findings support the concept that the elimination of infectious diseases should be approached as a complex adaptive public health system. Therefore, integrated intervention strategies that address multiple interconnected pathways may have a greater impact on measles elimination than single-sector interventions. Such perspectives are increasingly recognized as being essential to strengthening outbreak preparedness and resilient health systems [31].

Several strengths enhance the scientific contribution of this study. First, the analysis was conducted using a census of all 424 villages in Cirebon Regency rather than a sampled population, providing comprehensive coverage of the local epidemiological context and reducing the risk of selection bias. Second, the integration of routinely collected surveillance, immunization, nutritional, demographic, and health system data enabled a multidimensional assessment of measles elimination. Third, the application of SEM-PLS allowed for the simultaneous evaluation of both direct and indirect relationships among multiple determinants, providing a more comprehensive understanding of the complex pathways leading to measles elimination than would be possible using conventional regression approaches. Finally, the village-level analytical framework offers practical evidence for identifying localized transmission risks and supporting targeted elimination strategies at the subdistrict and village levels. Therefore, these findings have practical implications for strengthening decentralized elimination strategies in Indonesia and other developing countries [32].

This study also has some limitations that should be considered when interpreting the findings. First, the analysis was based on routinely collected secondary data, which may be affected by variations in data completeness and reporting quality across villages. Second, the ecological nature of the village-level analysis limits causal inference at the individual level and may be subject to ecological fallacy. Third, although the SEM-PLS model explained a substantial proportion of the variance in measles elimination (R² = 0.569), approximately 43% of the variance remained unexplained, suggesting that additional determinants, such as population mobility, vaccine hesitancy, viral genotypes, environmental conditions, and social and behavioral factors, may also contribute to elimination outcomes. Finally, the study was conducted in a single regency in Indonesia; therefore, caution should be exercised when generalizing the findings to other epidemiological settings with different health system characteristics.

Despite these limitations, the present findings provide important implications for measles elimination policy and practice. The integrated epidemiological model developed in this study indicates that sustainable measles elimination cannot be achieved through immunization programs alone but requires coordinated strengthening of surveillance-response systems, resilient local health systems, sustained nutritional improvement, and targeted interventions for vulnerable communities. By demonstrating how these determinants interact within a single predictive framework, this study offers practical evidence to support village-level risk stratification, early warning systems, and evidence-based resource allocation for measles elimination. Consequently, the proposed framework complements existing WHO measles elimination strategies by emphasizing that sustainable elimination requires not only high vaccination coverage but also integrated surveillance-response, strengthened local health system capacity, and risk-based interventions at the community level, thereby supporting evidence-based policy development in endemic settings [33]. Collectively, these findings demonstrate that measles elimination should be viewed not solely as a vaccination target but as the outcome of an integrated epidemiological system in which surveillance-response, health system capacity, population immunity, nutritional status, and community vulnerability interact to interrupt transmission and sustain elimination.

## Conclusion

This study developed a village-level epidemiological prediction model for measles elimination using SEM-PLS, showing that measles elimination is shaped by the integrated effects of surveillance-response performance, health system capacity, immunization coverage, nutritional status, community vulnerability, and disease transmission. Surveillance-response emerges as a key mediating mechanism linking upstream health system factors with downstream reductions in measles transmission, highlighting that sustainable elimination requires more than high vaccination coverage alone. By integrating epidemiological, surveillance, and health system determinants within a single predictive framework, this study provides a systems epidemiology perspective that supports village-level risk stratification, evidence-based resource allocation, and more adaptive subnational measles elimination strategies in Indonesia and other endemic settings.

## Public health implications

The findings have important implications for strengthening measles elimination strategies in Indonesia and other endemic settings. Surveillance-response systems should be prioritized as a core component of elimination efforts by enhancing early case detection, laboratory confirmation, epidemiological investigation, and rapid outbreak response, particularly through village-based surveillance. Elimination strategies should also integrate immunization, nutritional interventions, health system strengthening, and measures to reduce community vulnerability rather than relying solely on vaccination coverage. Furthermore, the proposed SEM-PLS prediction model provides a practical tool for village-level risk stratification, evidence-based resource allocation, and prioritization of interventions to support the implementation of more targeted and effective measles elimination strategies in resource-constrained settings.

## CRediT authorship contribution statement

**Agus Salim:** Writing – review & editing, Writing – original draft, Methodology, Formal analysis, Data curation, Conceptualization. **Hari Basuki Notobroto:** Supervision, Conceptualization. **Fariani Syahrul:** Supervision, Conceptualization. **Ade Nurlina:** Writing – review & editing, Writing – original draft, Supervision.

## Declaration of competing interest

The authors have no competing interests to declare.

## References

[1] World Health Organization. Framework for verifying elimination of measles and rubella; 2012. [Accessed February 13, 2026]. https://www.who.int/publications/i/item/framework-for-verifying-elimination-of-measles-and-rubella.

[2] World Health Organization (2025).

[3] Ekra K.D., Guehi C.H.H., Ahoussou E.M., Okoubo G. (2026). Prediction of measles elimination in Côte d’Ivoire by 2030. J Public Health Afr.

[4] Minta A.A., Ferrari M., Antoni S., Lambert B., Sayi T.S., Hsu C.H. (2024). Progress toward measles elimination — worldwide, 2000–2023. MMWR Morb Mortal Wkly Rep.

[5] World Health Organization. Strategic plan for measles and rubella elimination and sustenance in the WHO South-East Asia Region: 2024–2028. [Accessed February 13, 2026] https://www.who.int/publications/i/item/97892902294762024.

[6] O’Connor P., Masresha B., Pastor D., Musa N., Hagan J., Khanal S. (2024). Global status report for the verification of measles and rubella elimination, 2022. Vaccines (Basel).

[7] Cutts F.T., Dansereau E., Ferrari M.J., Hanson M., McCarthy K.A., Metcalf C.J.E. (2020). Using models to shape measles control and elimination strategies in low- and middle-income countries: a review of recent applications. Vaccine.

[8] Fatmi Nurhidayati, Irawati D., Dilaga MS. (2026). Measles trends, immunization coverage, and surveillance performance in Mataram city. Media of Health Research.

[9] Pampaka D., López-Perea N., Fernández-García A., Huertas-Zarco I., Castellanos-Martínez M., Villatoro-Bongiorno K. (2023). An interregional measles outbreak in Spain with nosocomial transmission, November 2017 to July 2018. Euro Surveill.

[10] Kamrujjaman M, Khondaker F, Sarker AA, Rivu NNK. Measles resurgence in Bangladesh, 2026: a situational analysis for urgent public health response. arXiv. 2026; arXiv:2604.25951. doi:10.48550/arXiv.2604.25951.

[11] Elliot A.J., Hughes H.E., Harcourt S.E., Smith S., Loveridge P., Morbey R.A. (2024). From fax to secure file transfer protocol: the 25-year evolution of real-time syndromic surveillance in England. J Med Internet Res.

[12] Ibrahim B.S., Usman R., Mohammed Y., Ahmed Z.D., Okunromade O., Abubakar A.A. (2019). Burden of measles in Nigeria: a five-year review of case-based surveillance data, 2012-2016. Pan Afr Med J.

[13] O’Connor P., Masresha B., Pastor D., Musa N., Hagan J., Khanal S. (2024). Global status report for the verification of measles and rubella elimination, 2022. Vaccines (Basel).

[14] Ansori ANM. (2026). Measles resurgence in Indonesia in 2026: post-pandemic immunity gaps and emerging public health challenges. Infect Dis Trop Med.

[15] Joffe M., Gambhir M., Chadeau-Hyam M., Vineis P. (2012). Causal diagrams in systems epidemiology. Emerg Themes Epidemiol.

[16] Dash G., Paul J. (2021). CB-SEM vs PLS-SEM methods for research in social sciences and technology forecasting. Technol Forecast Soc Change.

[17] Fernández Sánchez-Escalonilla S., Fernández-Escobar C., MÁ Royo-Bordonada (2022). Public support for the imposition of a tax on sugar-sweetened beverages and the determinants of such support in Spain. Int J Environ Res Public Health.

[18] Stronks K., Rod M.H., Rutter H., Rod NH. (2025). Towards a complex systems model of evidence for public health. BMJ Glob Health.

[19] Russell L., Uhre K.R., Lindgaard A.L.S., Degn J.F., Wetterslev M., Sivapalan P. (2021). Effect of 12 mg vs 6 mg of dexamethasone on the number of days alive without life support in adults with COVID-19 and severe hypoxemia. JAMA.

[20] Mulders M.N., Rota P.A., Icenogle J.P., Brown K.E., Takeda M., Rey G.J. (2016). Global measles and rubella laboratory network support for elimination goals, 2010–2015. MMWR Morb Mortal Wkly Rep.

[21] Patel M.K., Goodson J.L., Alexander J.P., Kretsinger K., Sodha S.V., Steulet C. (2020). Progress toward regional measles elimination — worldwide, 2000–2019. MMWR Morb Mortal Wkly Rep.

[22] Ferrari M.J., Grais R.F., Bharti N., Conlan A.J.K., Bjørnstad O.N., Wolfson L.J. (2008). The dynamics of measles in sub-Saharan Africa. Nature.

[23] Gao Y., Kc A., Chen C., Huang Y., Wang Y., Zou S. (2020). Inequality in measles vaccination coverage in the “big six” countries of the WHO South-East Asia region. Hum Vaccin Immunother.

[24] Gwela A., Mupere E., Berkley J.A., Lancioni C. (2019). Undernutrition, host immunity and vulnerability to infection among young children. Pediatr Infect Dis J.

[25] Isere E.E., Fatiregun AA. (2014). Measles case-based surveillance and outbreak response in Nigeria; an update for clinicians and public health professionals. Ann Ib Postgrad Med.

[26] Gong X., Zheng W., Lai S., Yin Z. (2024). Measles surveillance data analysis and serological survey in Quzhou, China, 2014–2024: an assessment of progress toward measles elimination. Front Med (Lausanne).

[27] Moss WJ., Griffin D.E., Oldstone M.B.A. (2009). Measles. Current Topics in Microbiology and Immunology.

[28] Skrip L.A., Townsend JP. (2019). Immunoepidemiology.

[29] Metcalf C.J.E., Ferrari M., Graham A.L., Grenfell BT. (2015). Understanding herd immunity. Trends Immunol.

[30] Bollen K.A., Pearl J., Morgan S.L. (2013). Handbook of Causal Analysis for Social Research.

[31] Salway S., Green J. (2017). Towards a critical complex systems approach to public health. Crit Public Health.

[32] Kong L., Wang J., Han W., Cao Z. (2016). Modeling heterogeneity in direct infectious disease transmission in a compartmental model. Int J Environ Res Public Health.

[33] Gastañaduy P.A., Banerjee E., DeBolt C., Bravo-Alcántara P., Samad S.A., Pastor D. (2018). Public health responses during measles outbreaks in elimination settings: Strategies and challenges. Hum Vaccin Immunother.

